# Genetic structure and life history are key factors in species distribution models of spiny lobsters

**DOI:** 10.1002/ece3.7043

**Published:** 2020-11-18

**Authors:** Sohana P. Singh, Johan C. Groeneveld, Sandi Willows‐Munro

**Affiliations:** ^1^ Oceanographic Research Institute Durban South Africa; ^2^ School of Life Sciences University of KwaZulu‐Natal Pietermaritzburg South Africa

**Keywords:** climate change, genetic differentiation, life history, MaxEnt, *Panulirus homarus*, species distribution modeling

## Abstract

**Aim:**

We incorporated genetic structure and life history phase in species distribution models (SDMs) constructed for a widespread spiny lobster, to reveal local adaptations specific to individual subspecies and predict future range shifts under the RCP 8.5 climate change scenario.

**Location:**

Indo‐West Pacific.

**Methods:**

MaxEnt was used to construct present‐day SDMs for the spiny lobster *Panulirus homarus* and individually for the three genetically distinct subspecies of which it comprises. SDMs incorporated both sea surface and benthic (seafloor) climate layers to recreate discrete influences of these habitats during the drifting larval and benthic juvenile and adult life history phases. Principle component analysis (PCA) was used to infer environmental variables to which individual subspecies were adapted. SDM projections of present‐day habitat suitability were compared with predictions for the year 2,100, under the RCP 8.5 climate change scenario.

**Results:**

In the PCA, salinity best explained *P. h. megasculptus* habitat suitability, compared with current velocity in *P. h. rubellus* and sea surface temperature in *P. h. homarus*. Drifting and benthic life history phases were adapted to different combinations of sea surface and benthic environmental variables considered. Highly suitable habitats for benthic phases were spatially enveloped within more extensive sea surface habitats suitable for drifting larvae. SDMs predicted that present‐day highly suitable habitats for *P. homarus* will decrease by the year 2,100.

**Main conclusions:**

Incorporating genetic structure in SDMs showed that individual spiny lobster subspecies had unique adaptations, which could not be resolved in species‐level models. The use of sea surface and benthic climate layers revealed the relative importance of environmental variables during drifting and benthic life history phases. SDMs that included genetic structure and life history were more informative in predictive models of climate change effects.

## Graphical abstract

We constructed present‐day species distribution models (SDMs) for the widespread spiny lobster *Panulirus homarus* and individually for the three genetically distinct subspecies of which it comprises. The SDMs were used to reveal local adaptations specific to individual subspecies and to predict future range shifts under the RCP 8.5 climate change scenario. Our approach included both sea surface and benthic (sea floor) climate layers to recreate discrete influences of these habitats during the drifting larval and benthic adult life history phases. Our analyses confirmed that accounting for genetic partitioning in models of widespread marine species can reveal unique local adaptations, which were unresolved in models that excluded the genetic information.

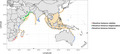

## 1 INTRODUCTION

Species distribution models (SDMs) are popular numerical methods which correlate geo‐located species occurrence data with environmental datasets to infer habitat suitability (Elith & Leathwick, 2009). SDMs have many applications in applied ecology and biogeography, such as predicting suitable sites for species, range shifts, and spread of invasive species and predicting future distribution patterns as a result of climate change (Melo‐Merino et al., 2020). SDMs have been used extensively in terrestrial ecosystems. Their use in marine ecosystems has increased significantly in recent years, enabled by the rapid growth of remote sensing technology and increasing availability of high‐resolution marine environmental data available on global databases (reviewed by Melo‐Merino et al., 2020; Robinson et al., 2017). Even so, using SDMs to model distributions in marine environments remains complex, because of the dynamic nature of physical and biological variables in these systems (Palumbi et al., 2019; Robinson et al., 2011). In marine environments, species–environment relationships occur at multiple spatial and temporal scales, but in most cases environmental data are restricted to sea surface and benthic layers, with limited data for midwater habitats (Assis et al., 2017). Life history phases of marine invertebrates often inhabit different discrete habitats, as drifting larvae dispersing in sea surface or midwater layers, and as juveniles and adults in benthic environments (Robinson et al., 2011). Water movements (ocean currents, eddies, fronts) are highly variable and together with larval duration and behavior determine the spatial reach of dispersal processes (Butler et al., 2011). The combination of dispersal processes, location of benthic habitats, and life history traits regulates recruitment, survival, and reproduction, and ultimately determines geographic distribution patterns (Soberon & Peterson, 2005).

Exposure to habitat changes can strongly influence the phenotypic plasticity of a species, directly contributing to population fitness, and adaptive genetic variation and evolution (Reed et al., 2011). Local adaptation typically occurs when populations near the edge of a species range develop tolerance to more extreme environmental conditions, sometimes accompanied by a loss of tolerance to formerly suitable conditions (Huey & Hertz, 1984). Over time, locally adapted populations may become genetically differentiated, giving rise to genetically structured populations across their geographic range. Local adaptation and genetic structure within widespread marine invertebrates have been documented for planktonic taxa (reviewed in Sanford & Kelly, 2011), spiny lobster (Gaeta et al., 2020; Lavery et al., 2014; Singh et al., 2018; Truelove et al., 2017), crabs (van Tienderen & van der Meij, 2017), oysters (Burford et al., 2014), and sea urchins (Carreras et al., 2020). Recent studies have shown that incorporating local adaptation and genetic structure in SDMs can be informative in predictive models of climate change effects (Cacciapaglia & van Woesik, 2018; Hällfors et al., 2016).

Spiny lobsters (Palinuridae) support economically valuable fisheries wherever they are found. Lobster species are often regional icons, and they are among the most researched animals on earth (Phillips, 2013). Spiny lobsters occupy a trophic level as benthic consumers and are essential to the function and maintenance of reef ecosystems where they occur at high densities (Briones‐Fourzán & Lozano‐Álvarez, 2015). Spiny lobsters are highly fecund, and after hatching, larvae drift in ocean currents for several months to years before settling on the seafloor (George, 2005). Larvae can be dispersed over vast distances by prevailing ocean currents (Chiswell et al., 2003; Groeneveld et al., 2012) or retained near their origin by submesoscale processes and larval behavior (Butler et al., 2011; Chiswell & Booth, 1999; Kough et al., 2013; Singh et al., 2019). The effects of environmental change on spiny lobster distribution and abundance have been well‐documented for several species, particularly in relation to fisheries management (Caputi et al., 2010, 2013; Chávez & García‐Córdova, 2011; Cockcroft et al., 2008).

Here, we chose the scalloped spiny lobster *Panulirus homarus* (Linnaeus, 1758) as a model organism for testing SDM habitat suitability predictions at an ocean‐wide scale. *P. homarus* is widespread in the tropical and subtropical Western Indo‐Pacific (Chan, 2010; Holthuis, 1991) and inhabits shallow coastal reef patches exposed to waves and currents. Females release larvae with a prolonged (4–6 months) drifting dispersal phase (George, 2005; Phillips & Booth, 1994). The long drifting larval phase is susceptible to the effects of climate change, especially variability in the strength and direction of ocean currents and water temperature (Singh et al., 2018, 2019). *P. homarus* comprises three subspecies based on geography and morphological characteristics (Berry, 1974). An olive‐green megasculpta form (*P. h. megasculptus*) occurs in the NW Indian Ocean (coastal Somalia and Arabian Sea), a red megasculpta form (*P. h. rubellus*) occurs in the SW Indian Ocean (Mozambique, eastern South Africa, and SE Madagascar), and the nominotypical dark‐green microsculpta form (*P. h. homarus)* is widespread in the Western and Central Indo‐Pacific. The divergence of these three subspecies is supported by genetic data. Phylogenetic and population genetic studies have confirmed that the *P. h. rubellus* morphotype is a separately evolving lineage (Farhadi et al., 2017; Lavery et al., 2014; Singh et al., 2017). Farhadi et al. (2013, 2017), have also shown molecular evidence that *P. h. megasculptus* is diverging from *P. h. homarus*.

The aims of this study were to investigate the effects of genetic partitioning on predictions of current and future areas of habitat suitability for the commercially important spiny lobster *Panulirus homarus*, using SDMs. Genetic and oceanographic barriers derived from Singh et al. (2018) were incorporated in the SDMs, and the results were contrasted with SDMs that treated *P. homarus* as a single homogenous unit (i.e., all subspecies combined). We took both larval dispersal and benthic life history phases into account by modeling sea surface and benthic environmental data layers. SDMs were used to forecast future changes in suitable habitats for *P. homarus* (to the year 2,100) under a maximum greenhouse gas emission scenario: representative concentration pathways (RCP 8.5), which depicts a worst‐case scenario for the rise in global emissions (IPCC 2013). RCP 8.5 assumes that by the year 2,100, the radiative forcing level will reach 8.5 W/m^2^, sea level will have increased by >90 cm, and seawater temperature will be approximately 3°C warmer. We predicted that SDMs that incorporated genetic partitioning would reveal local adaptations that could not be discerned from models that treated *P. homarus* as a homogenous unit and that predicted shifts in the geographic distribution of the three subspecies under the RCP 8.5 climate change scenario would be influenced by local adaptations.

## 2 METHODS

### 2.1 Study region

The Western Indo‐Pacific biogeographic realm (Spalding et al., 2007) covers the western and central parts of the Indian Ocean, including the east coast of Africa, Red Sea, Gulf of Aden, Persian Gulf, Arabian Sea, Bay of Bengal, and Andaman Sea, as well as the coastal waters surrounding Madagascar, the Seychelles, Comoros, Mascarene Islands, Maldives, and Chagos Archipelago. *P. homarus* also occurs along the western edge of the Central Indo‐Pacific, including Indonesia, Thailand, Taiwan, northern Australia, and as far to the east as Japan (Holthuis, 1991). The study region included the entire known distribution range of *P. homarus*, extending across tropical and subtropical latitudes.

### 2.2 Environmental and geographic occurrence data

Both larval dispersal and benthic life history phases were taken into account by modeling sea surface and benthic environmental data layers. Monthly averaged climatic variables (current velocity, water temperature, and salinity) for the present sea surface (to model larval habitat suitability) and benthic (to model adult habitat suitability) layers (2000–2014) as well as forecasted data layers for the RCP 8.5 climate change scenarios for the year 2,100 were downloaded from Bio‐ORACLE (Ocean Rasters for Analysis of Climate and Environment, http://www.oracle.ugent.be; Assis et al., 2017; Tyberghein et al., 2012) at 5 arcmin spatial resolution (9.2 km). Predictor variables were chosen if present and future projections were available for the study area and for both surface and benthic layers (Table 1). A variance inflation factor analysis (VIF, Marquardt, 1970) was carried out by regressing each predictor variable against the others to assess potential collinearity. The VIF analysis was performed in the R package *usdm* (Naimi, 2012), and variables with a VIF > 10 (an indication of collinearity) were excluded from subsequent analyses (Chatterjee & Hadi, 2006).

**TABLE 1:** The receiver operating characteristic area under the curve (AUC) and standard deviation (*SD*), percentage contribution, and permutation importance for surface and benthic predictor variables for *Panulirus homarus* subspecies combined and for individual subspecies

Variable	Present			RCP 8.5		
	% Contribution	Permutation Importance	AUC ± *SD*	% Contribution	Permutation Importance	AUC ± *SD*
*P. homarus* subspecies combined						
Mean surface current velocity	23	17.8	0.96 ± 0.006	26	24.8	0.95 ± 0.005
Maximum surface current velocity	6.6	2.5		6	2.3	
Minimum surface current velocity	3.1	1.3		3	1.5	
Range surface current velocity	7.8	2.6		8.1	5	
Long‐term maximum surface temperature	**19**	**40**.**7**		**17**.**4**	**25**.**4**	
Range surface temperature	10.5	5.4		10	8.5	
Maximum surface salinity	**18.9**	**13.6**		**19.8**	**15.1**	
Range surface salinity	11.1	16.1		9.7	17.4	
Mean benthic current velocity	5.6	5.8	0.99 ± 0.001	6	4.1	0.99 ± 0.002
Maximum benthic current velocity	8.6	0.3		8.4	1.1	
Minimum benthic current velocity	15.9	11.9		13.1	4.3	
Range benthic current velocity	2	1.1		3.8	0.6	
Minimum benthic temperature	**16.3**	**69.8**		**24**	**78.2**	
Range benthic temperature	18	1.9		5.1	1.8	
Long‐term maximum benthic salinity	7.9	1.6		7.7	4.1	
Range benthic salinity	**25.8**	**7.6**		**31.9**	**5.8**	
*P. h. homarus*						
Mean surface current velocity	24.2	7.6	0.95 ± 0.018	33.7	12.7	0.95 ± 0.016
Maximum surface current velocity	3.2	1.1		1.1	0.2	
Minimum surface current velocity	3.9	1.3		3.6	0.6	
Range surface current velocity	4.2	2.5		3.3	0.9	
Long‐term maximum surface temperature	10	21.8		5.3	15.9	
Range surface temperature	6.9	7.3		7.6	8.5	
Maximum surface salinity	0.6	2.9		0.9	0.7	
Range surface salinity	**47**	**55.5**		**44.5**	**60.5**	
Mean benthic current velocity	11.5	19.8	0.99 ± 0.004	12.7	11.6	0.99 ± 0.002
Maximum benthic current velocity	13.7	3.6		11.9	3	
Minimum benthic current velocity	**24.6**	**10.6**		**27.4**	**15.3**	
Range benthic current velocity	4.3	1.3		2	2.6	
Minimum benthic temperature	**8.9**	**38.4**		**7.3**	**30.2**	
Range benthic temperature	7.3	3.6		0.9	8.9	
Long‐term maximum benthic salinity	0.1	0.7		0.8	1.3	
Range benthic salinity	**29.6**	**22**		**37.1**	**27.1**	
*P. h. megasculptus*						
Mean surface current velocity	2.2	0.7	0.99 ± 0.0	3.7	0.2	0.99 ± 0.001
Maximum surface current velocity	5	0		10.1	0.1	
Minimum surface current velocity	1.5	0.4		1.7	0.4	
Range surface current velocity	4.8	0.1		3.3	0.1	
Long‐term maximum surface temperature	19.4	38.6		19.2	45.9	
Range surface temperature	15.3	1.3		14.2	1	
Maximum surface salinity	**50.1**	**58**		**44**	**50.7**	
Range surface salinity	1.6	0.9		3.9	1.5	
Mean benthic current velocity	0.4	1.9	0.99 ± 0.001	0.6	0.3	0.99 ± 0.001
Maximum benthic current velocity	**22.8**	**18.9**		**22.1**	**10**	
Minimum benthic current velocity	9.3	5.5		11.4	7.3	
Range benthic current velocity	1.3	3.4		2	2.5	
Minimum benthic temperature	6.5	17.5		**7.1**	**24.7**	
Range benthic temperature	**36**.**4**	**2**		**32**.**1**	**2**.**8**	
Long‐term maximum benthic salinity	**12.1**	**47.4**		**10.3**	**26.4**	
Range benthic salinity	11.3	3.5		14.5	25.9	
*P. h. rubellus*						
Mean surface current velocity	**36.2**	**29.1**	0.99 ± 0.0	**47.1**	**26.5**	0.99 ± 0.001
Maximum surface current velocity	31.4	8.2		26.3	11.5	
Minimum surface current velocity	0.6	0.7		1.8	0.4	
Range surface current velocity	7.9	0.3		5.1	0.3	
Long‐term maximum surface temperature	**16.4**	**57.5**		**11.6**	**35.9**	
Range surface temperature	6.6	3.7		6.7	24.6	
Maximum surface salinity	0.2	0.4		0	0.1	
Range surface salinity	0.7	0.2		1.3	0.6	
Mean benthic current velocity	**42.3**	**0.4**	0.99 ± 0.0	**61.2**	**1.8**	0.99 ± 0.0
Maximum benthic current velocity	1.3	0.2		1.2	0.3	
Minimum benthic current velocity	7.8	0.2		8.1	0.6	
Range benthic current velocity	1.5	0		4	0.2	
Minimum benthic temperature	**20.9**	**98.8**		**12.9**	**95.6**	
Range benthic temperature	20.2	0.2		3.1	0.3	
Long‐term maximum benthic salinity	0	0		0.3	0.1	
Range benthic salinity	6	0.2		9.3	1.1	

Note

Important predictor variables are highlighted in bold. Under scenario RCP 8.5, the mean and likely global warming increase by 2,081–2,100 is predicted to be 3.7°C (2.6–4.8°C). Global mean sea level (m) increase projection is 0.63 m (0.45–0.85 m) under RCP 8.5 (IPCC Fifth Assessment Report 2014).

**FIGURE 1:**
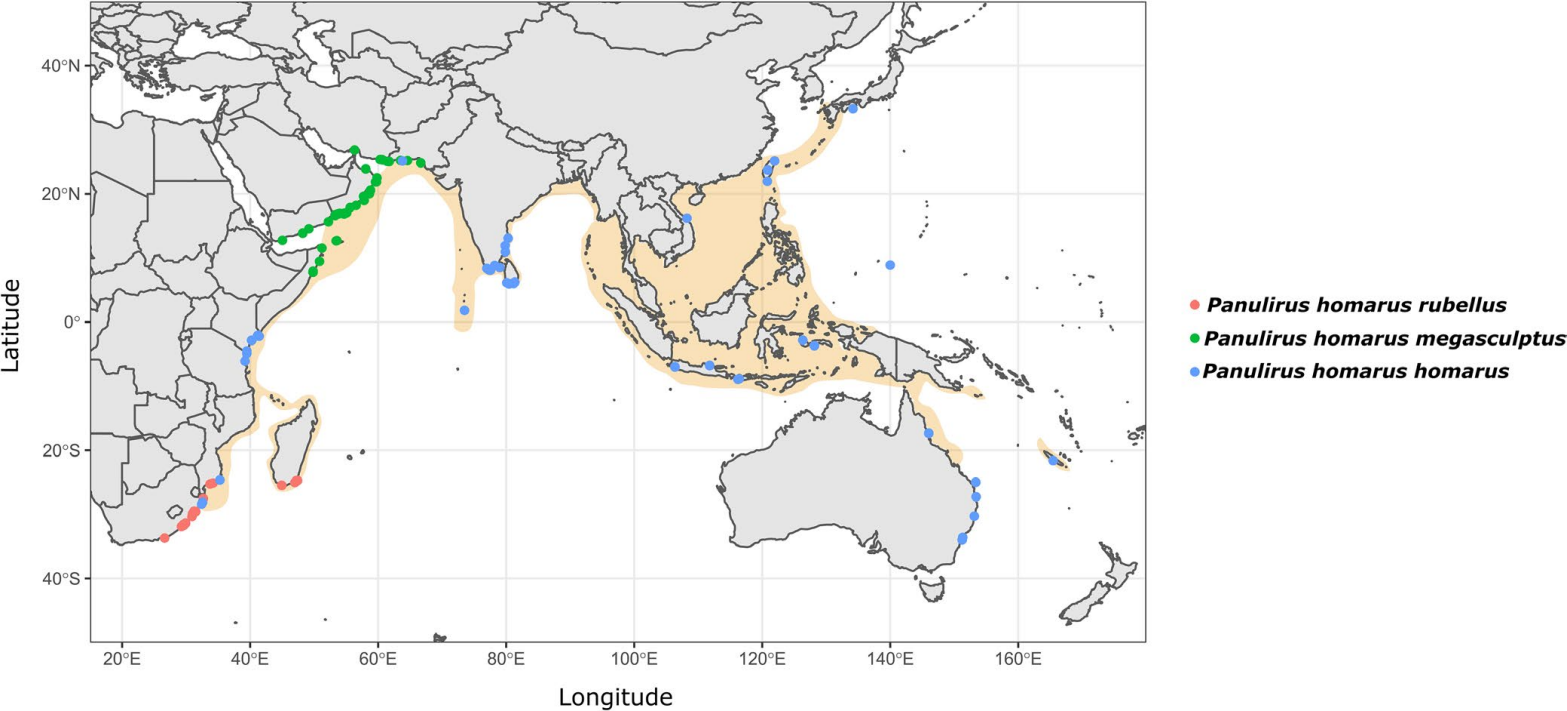
*Panulirus homarus* subspecies occurrence points, with the present‐day known distribution range throughout the Indo‐West Pacific highlighted in orange

Georeferenced occurrence data for *P. homarus* were obtained from the Global Biodiversity Information Facility (www.gbif.org) and the Ocean Biogeographic Information System (www.iobis.org). Occurrence data obtained from the published literature were also added (Lavery et al., 2014; Reddy et al., 2014; Singh et al., 2018; Singh et al., 2019). Lobster fishing locations (described by Al‐Breiki et al., 2018; Al Marzouqi, Al‐Nahdi, et al., 2008; Al Marzouqi, Groeneveld, et al., 2008; Amali & Wulan Sari, 2020; Fielding & Mann, 1999; Hariri et al., 2000; Jong, 1993; Mashaei & Rajabipour, 2002, 2003; Priyambodo et al., 2017; Sanders & Bouhlel, 1984; Senevirathna & Munasinghe, 2014; Sultana et al., 2009; Thangaraja et al., 2015) were used to derive additional occurrence data after validation by plotting representative locations on Google Earth (earth.google.com/web/). The occurrence data consisted of 114 unique occurrence points throughout the Western Indo‐Pacific and the western part of the Central Indo‐Pacific (Figure 1). Grouping of the populations by subspecies was informed by morphological identification of specimens (where available), and genetic and geographic information (Lavery et al., 2014; Singh et al., 2018, 2019).

Principal component analyses (PCAs) implemented in the R package *FactoMineR* (Lê et al., 2008) using all present surface and benthic environmental variables were conducted to examine whether the occurrence data of each subspecies are linked to environmental variables.

### 2.3 SDM construction and forecasting

The machine‐learning method MaxEnt 3.4.1 (Phillips & Dudík, 2008) was used to develop present‐day and forecasting SDMs (based on the RCP 8.5 climate change scenario) for *P. homarus* (subspecies combined) and for each subspecies separately. SDMs were based on marine sea surface and benthic climate layers (Table 1). The MaxEnt models were generated using 10 bootstrap replicate runs for each model and with 10,000 background points. During optimization 25% of the data were used as test data and 75% as training data. The final models were run using all the data (100%). A logistic output was chosen, and the average of the 10 replicates was used for further analyses. The performance of each model was evaluated using the receiver operating characteristic area under the curve (AUC, Fielding & Bell, 1997). A permutation approach and jackknife tests were used to assess the importance of the predictor variables in predicting the species distribution. Initial calibration models were run with all 18 climate variable layers, but subsequent analyses were only conducted with layers which were not excluded in the VIF analysis (see above).

GRASS GIS v. 7.0.4 (Neteler et al., 2012) was used to import raster maps of habitat suitability from MaxEnt and to calculate the area (km^2^) of habitat suitability. Pixels on the raster maps were classified into probabilities: not suitable (0–0.39), low suitability (0.4–0.59), medium suitability (0.6–0.79), and high suitability (0.80–1.0). The inferred distribution range was constrained to the eastern hemisphere (0–180°E) and to latitudes between 50°N and 50°S, to exclude ocean regions considered unlikely to be naturally colonized by *P. homarus*. The Mediterranean Sea was also excluded from the present‐day SDMs because *P. homarus* has not been recorded there.

## 3 RESULTS

### 3.1 PCA outputs

**FIGURE 2:**
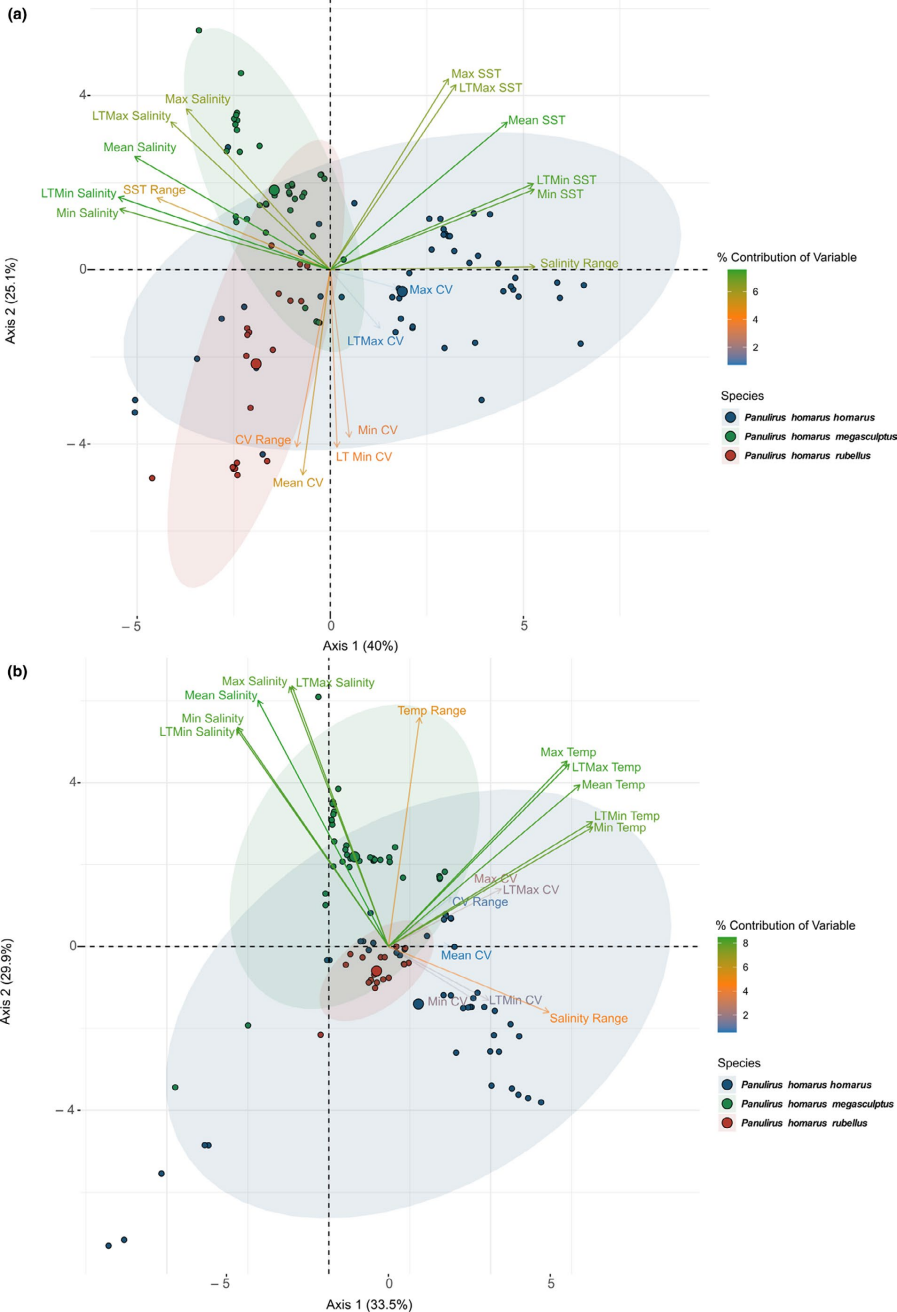
PCA plot of *Panulirus homarus* subspecies using all (a) surface and (b) benthic predictor variables. The ellipses represent 95% confidence intervals of subspecies groupings. The percentages by axes indicate how much variation is explained by the principal components. CV, current velocity; LT, long‐term

For surface environmental variables, the first PCA axis explained 40%, and the second axis 25% of subspecies niche suitability (Figure 2a). Variables contributing most to the difference between subspecies on the first axis were long‐term minimum salinity, minimum salinity, and salinity range. On the second axis, mean current velocity, maximum SST, and long‐term maximum SST contributed most to the observed variation. For benthic environmental variables, the first PCA axis explained 34% of the difference in niche suitability, with SST (minimum, long‐term maximum, and maximum) contributing most (Figure 2b). The second axis explained 30%, with salinity (long‐term maximum, maximum, and mean salinity) contributing most to the observed differences. Overall, niche suitability of *P. h. megasculptus* was best explained by salinity variables, *P. h. rubellus* by current velocity variables, and *P. h. homarus* by SST.

### 3.2 Sea surface variables

The present‐day and RCP 8.5 SDMs using sea surface predictor variables for subspecies combined and for individual subspecies were robust, with all AUC values above 0.9 (Table 1). In the present‐day model of combined subspecies, mean current velocity (23%), long‐term maximum temperature (19%), and maximum salinity (19%) explained most of the variance (Table 1). These three variables were also the most important in the RCP 8.5 model of combined subspecies, explaining 26%, 17%, and 20% of variance, respectively. The long‐term maximum temperature had the highest permutation importance in both models.

When modeled as separate subspecies, salinity range explained 47% of the present‐day variance for *P. h. homarus* and 45% in the RCP 8.5 model (Table 1). Mean current velocity explained the bulk of the remaining variance in present‐day (24%) and RCP 8.5 (34%) models. Salinity had the highest permutation importance in both models.

For *P. h. megasculptus*, long‐term maximum salinity explained 50% and 44% of variance in the present‐day and RCP 8.5 models (Table 1). Long‐term maximum temperature explained 19% for the present‐day and RCP 8.5 models. Maximum salinity had the highest permutation importance in both models.

For *P. h. rubellus*, mean current velocity explained 36% of variance in the present‐day model and 47% in the RCP 8.5 model, but the long‐term maximum surface temperature had the highest permutation importance in both models (Table 1). Maximum current velocity explained 31% and 26% of variance in present‐day and RCP 8.5 models.

### 3.3 Benthic variables

The present‐day and RCP 8.5 SDMs based on benthic climate layers were robust for the three subspecies combined and for individual subspecies, with all AUC values above 0.9 (Table 1). For subspecies combined, salinity range explained 26% of variability in present‐day and RCP 8.5 models, and minimum temperature (16% and 24%) and maximum current velocity (16% and 13%) explained most of the remainder. Minimum temperature had the highest permutation importance in both models.

When modeled as individual subspecies, salinity range explained 30% of variance in present‐day and 37% in RCP 8.5 models for *P. h. homarus*, followed by minimum current velocity (25% and 27%). Minimum temperature had a high permutation importance in both models (Table 1). For *P. h. megasculptus*, temperature range explained 36% of variance in the present‐day model and 32% in the RCP 8.5 model, and maximum current velocity explained most of the remainder (23% and 22%). Long‐term maximum salinity had the highest permutation importance in the present‐day model and in the RCP 8.5 model. For *P. h. rubellus*, mean current velocity explained 42% of variance in the present‐day model and 61% in the RCP 8.5 model, but minimum temperature had the highest permutation importance in both models.

### 3.4 Species distribution probabilities for present‐day and the RCP 8.5 climate change scenario

**FIGURE 3:**
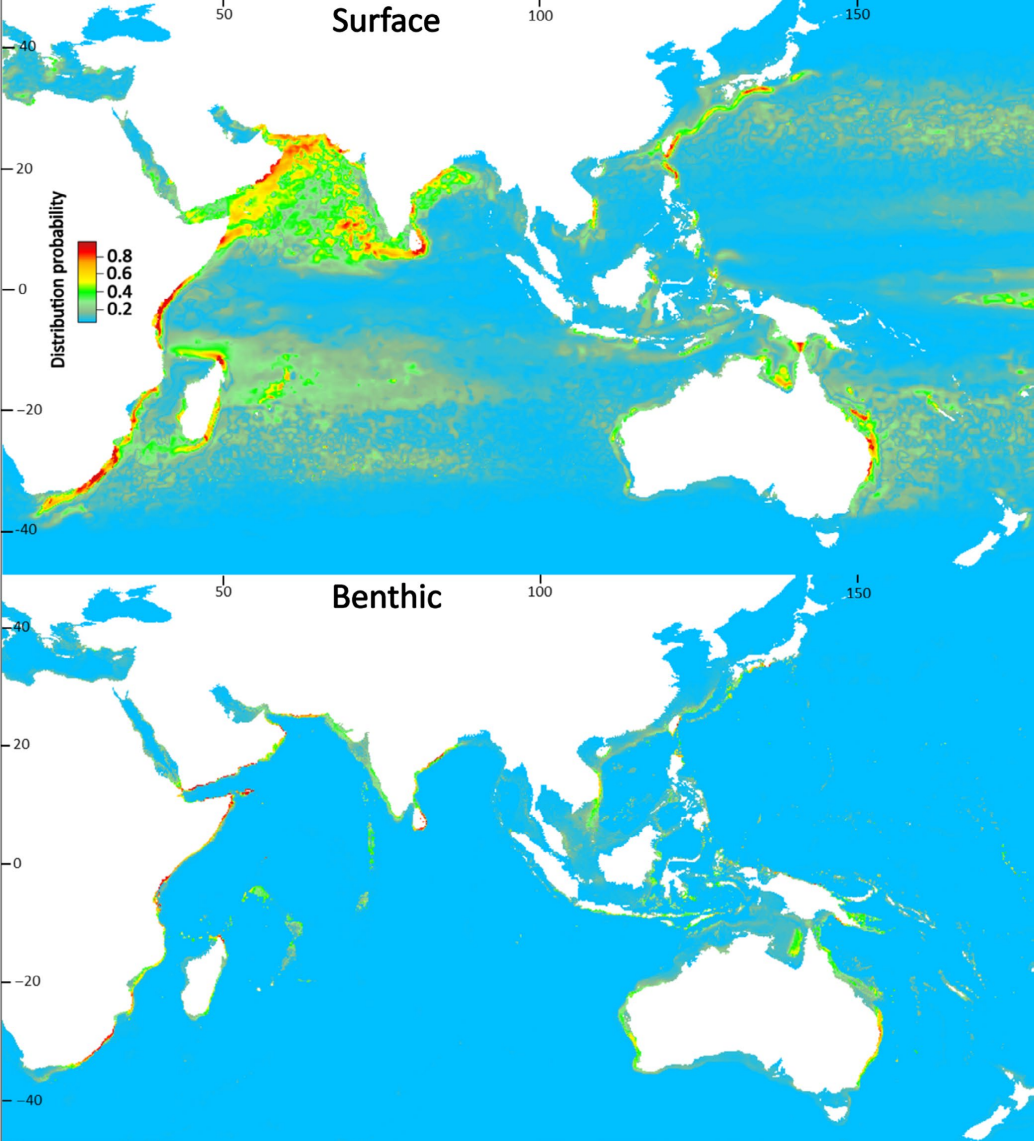
Surface and benthic habitat suitability maps for *Panulirus homarus* modeled as three subspecies combined for the present‐day. Highly suitable habitat in red

**FIGURE 4:**
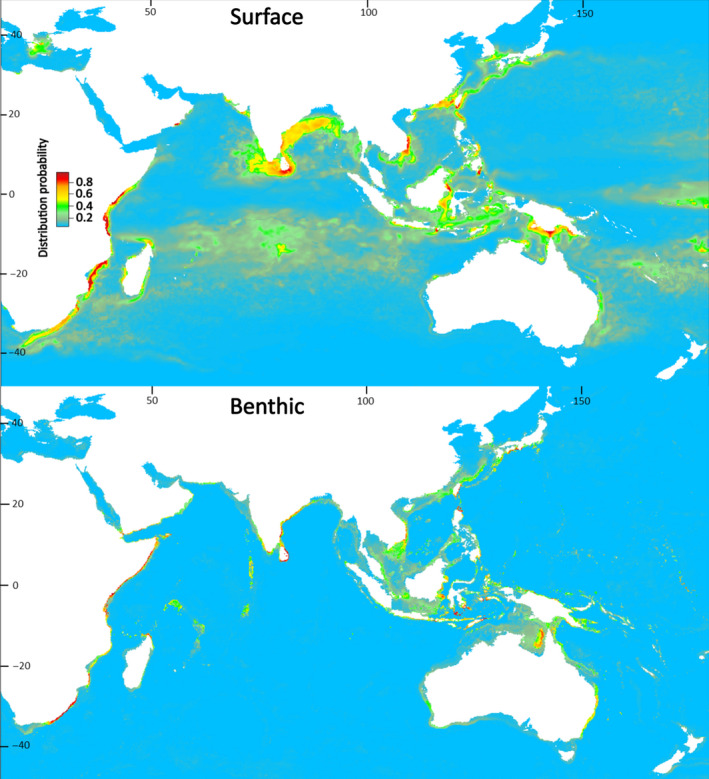
Surface and benthic habitat suitability maps for the *Panulirus homarus homarus* subspecies for the present‐day. *P. h. homarus* has the broadest distribution range of the three subspecies, overlapping the distributions of *P. h. rubellus* in the SW and *P. h. megasculptus* in the NW Indian Ocean. Highly suitable habitat in red

**FIGURE 5:**
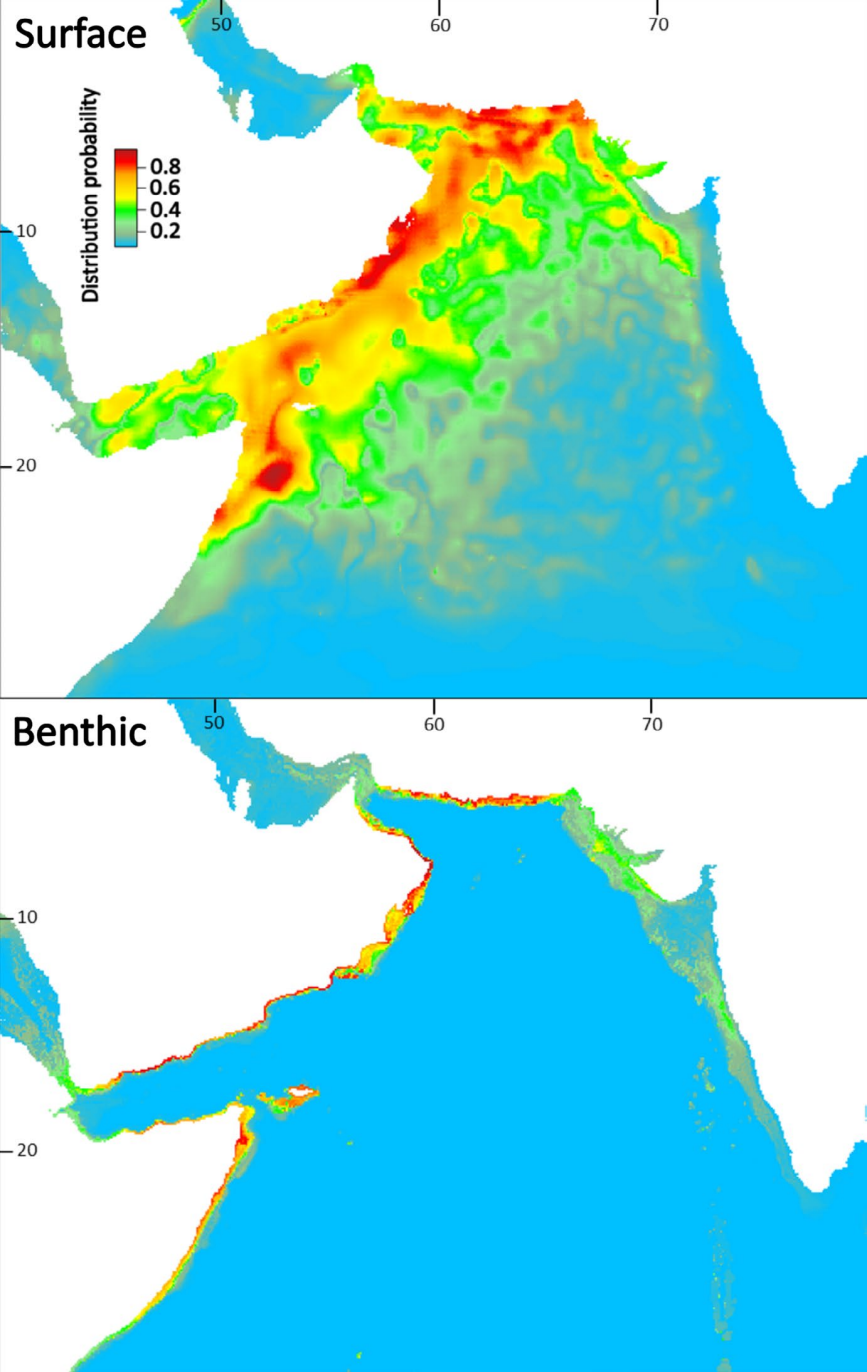
Surface and benthic habitat suitability maps for the *Panulirus homarus megasculptus* subspecies for the present‐day. Benthic (juvenile and adult) *P. h. megasculptus* populations are presently restricted to the NW Indian Ocean (Arabian Sea and coastal Somalia)

**FIGURE 6:**
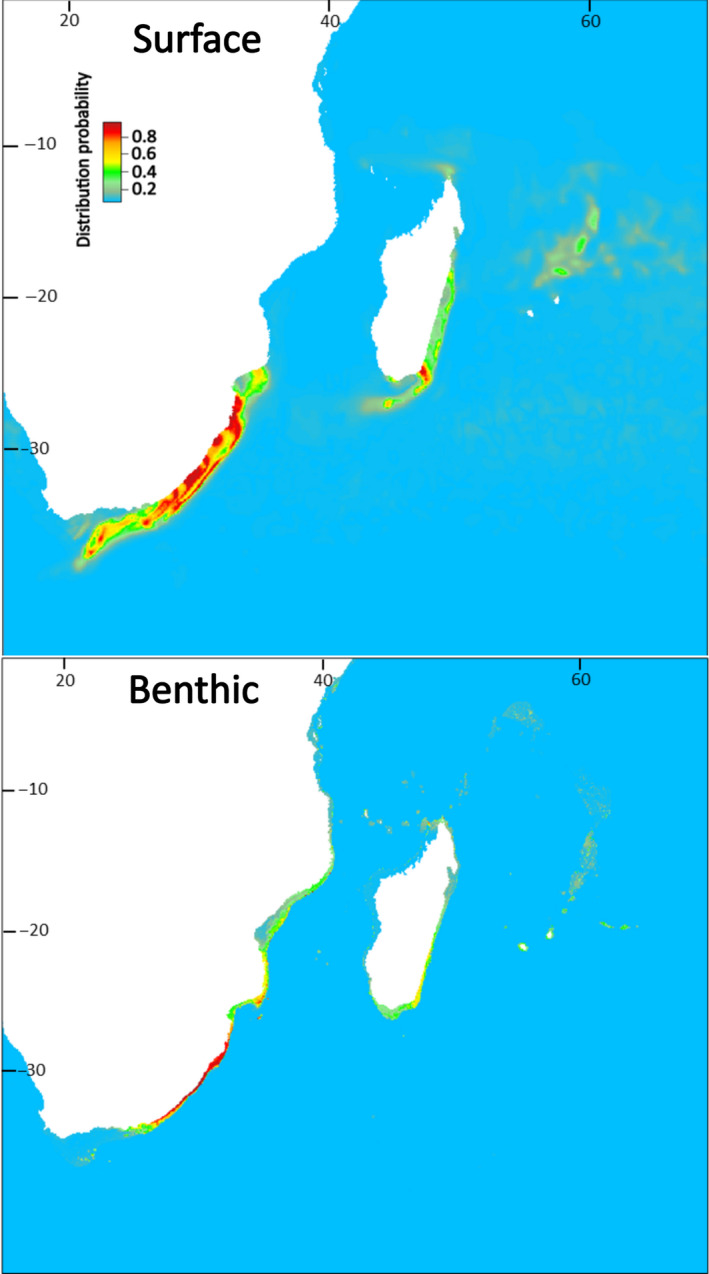
Surface and benthic habitat suitability maps for the *Panulirus homarus rubellus* subspecies for the present‐day. Benthic (juvenile and adult) *P. h. rubellus* populations are presently restricted to the SW Indian Ocean (Mozambique, eastern South Africa, SE Madagascar)

Present‐day estimates of highly suitable sea surface area for larval dispersal phases were substantially larger than the size of benthic areas for juvenile and adult *P. homarus* (subspecies combined), and for individual subspecies (Table 2). Benthic habitat areas were spatially contained within estimated surface habitat areas. Suitable benthic habitat areas were closely aligned with shallow coastal waters (Figures 3, 4, 5, 6), matching known seafloor habitats of *P. homarus* (Holthuis, 1991). Sea surface habitats extended far beyond nearshore continental shelf waters, and when including medium suitability probabilities, they sometimes extended to basin‐wide scales. Overall, the present‐day high and medium distribution probabilities depicted robust likely habitat distributions, matching well to known distribution patterns of *P. homarus*.

**TABLE 2:** Highly suitable habitat areas (km^2^ ± *SD*) estimated by sea surface and benthic SDMs and proportional change under the RCP 8.5 climate change scenario, relative to the present‐day area (1.00)

	Subspecies combined	*Panulirus homarus homarus*	*Panulirus homarus megasculptus*	*Panulirus homarus rubellus*
Surface SDMs				
Est. present‐day area ± *SD* (km^2^*1,000)	310 ± 15	264 ± 34	116 ± 17	96 ± 26
Relative change under RCP 8.5	1.08	1.08	0.92	0.67
Benthic SDMs				
Est. present‐day area (km^2^*1,000)	128 ± 18	207 ± 42	58 ± 15	32 ± 3
Relative change in suitable habitat area	0.80	0.60	0.79	0.66

Sea surface SDMs under the RCP 8.5 climate change scenario predicted moderate future expansions in highly suitable habitat areas for dispersing larvae of subspecies combined (+8%) and for *P. h. homarus* (+8%) but contractions for *P. h. megasculptus* (−8%) and *P. h. rubellus* (−33%) (Table 2). Benthic SDMs predicted highly suitable habitat contractions of between 20% for subspecies combined and as much as 40% for *P. h. homarus* under the RCP 8.5 scenario (Table 2).

## 4 DISCUSSION

At the onset of this study, we predicted that SDMs based on genetically partitioned subspecies of *P. homarus* would reveal local adaptations that cannot be discerned when they are modeled as a single species and that shifts in geographic distribution under the RCP 8.5 climate change scenario would be influenced by unique adaptations. Both predictions were strongly supported by the analyses presented. Sea surface SDMs for the three subspecies combined attributed variance roughly equally to current velocity (40%), water temperature (30%), and salinity variables (30%), but individual models could identify the relative importance of variables unique to each subspecies. A similar pattern was observed for benthic SDMs: Roughly equal variance attributed to current velocity (32%), water temperature (34%), and salinity variables (34%) for combined subspecies, but specific variables became relatively more important in models of individual subspecies. Overall, our findings aligned well with the theory that natural selection drives populations exposed to more extreme environmental conditions to adapt to become better suited to its local conditions than other members of the same species (Bell & Collins, 2008; Huey & Hertz, 1984).

*Panulirus homarus rubellus* inhabits a narrow continental shelf flanked by the upper reaches of the strong western boundary Agulhas Current. Drifting larvae released in this environment can either be retained over the narrow shelf by submesoscale processes, dispersed downstream along the coast, or become entrained in the Agulhas Current and presumably lost offshore (Singh et al., 2019). Recent gene flow and population genetic structure analyses supported a hypothesis that some larvae are retained nearshore by lee eddies and counter currents, and that net gene flow direction was toward the southwest (Singh et al., 2018, 2019). In the present study, the sea surface SDM for *P. h. rubellus* attributed nearly 80% of variance to current velocity variables, and the benthic SDM, some 55%. The SDM results therefore agree with gene flow and oceanographic information, confirming that *P. h. rubellus* is adapted primarily to survival in a strong ocean current regime. Two other spiny lobster species occurring in the same region have similarly adapted to survival in strong ocean currents, by undertaking benthic migrations to redress downstream dispersal of larvae (Groeneveld, 2002; Groeneveld & Branch, 2002; Santos et al., 2014).

*Panulirus homarus megasculptus* inhabits the NW Indian Ocean but is excluded from the Red Sea and Persian Gulf, potentially because larvae are unable to tolerate high salinity water which forms in enclosed water bodies where evaporation exceeds precipitation (Jha et al., 2010; Kumar & Prasad, 1999). The hypersaline water then exits the Red Sea into the Gulf of Aden and the Persian Gulf into the Sea of Oman (Kumar & Prasad, 1999). We suggest that these areas form biogeographic transition zones to the successful dispersal of *P. h. megasculptus* larvae, thus explaining the importance of maximum salinity in the sea surface SDM (50% of variance explained). Khvorov et al. (2012) found that phyllosoma larvae (most likely *P. h. megasculptus*) were nearly twice as abundant in the Arabian Sea than in the Sea of Oman, thus supporting a transition from high to low habitat suitability. In contrast to the surface SDM, salinity explained only a minor portion of variance in the benthic SDM (23%), with bottom temperature explaining most variance (43%). Water temperature in the coastal Arabian Sea fluctuates considerably because of opposing influences of cold‐water upwelling cells and solar heating (Marra & Barber, 2005). Tolerance to sudden changes in water temperature would therefore be an adaptive advantage to benthic species unable to move away. *P. h. megasculptus* is therefore adapted to tolerate a wide range of water temperature in benthic and salinity in drifting larval phases.

*Panulirus homarus homarus* has a more equatorial distribution than the other two subspecies, but also occurs in sympatry with *P. h. rubellus* in the SW Indian Ocean and *P. h. megasculptus* in the NW Indian Ocean, albeit at much lower abundance. Its occurrence throughout the distribution range of all *P. homarus* subspecies, in tropical and subtropical latitudes, and in areas with variable current velocities, then suggests that it is adapted to a wide range of environmental variables. In the surface SDM, most variance was attributed to salinity range (47%), and in the benthic SDM, current velocity variables explained most variance (54%). Water temperature explained 17% of variance in the sea surface SDM, and 16% in the benthic SDM, and in both cases, it had high permutation importance. The SDMs therefore support adaptation to a broader range of variables in *P. h. homarus* than in the other two subspecies.

Although statistically robust, the SDM outputs may have been affected by three key assumptions: that all areas were sampled equally (Araujo & Guisan, 2006); that geo‐located occurrence data were accurate; and that sea surface layers could explain pelagic habitats without including midwater layers (Bentlage et al., 2013). For benthic life history phases, occurrence records obtained from regional databases (GBIF and OBIS), the published literature and locational data from commercial fisheries were verified by plotting on Google Earth (earth.google.com/web/), and the final occurrence dataset represented the known distribution of *P. homarus* well, including geographic information on subspecies distribution patterns (see Figure 1).

For drifting larval phases, occurrence records were restricted to verified adult habitats—the point of hatching from eggs carried by female lobsters. No geo‐located data were available to describe the oceanic distribution of drifting phyllosoma larvae. Nevertheless, a process‐oriented Lagrangian particle dispersal model developed for *P. homarus* (Singh et al., 2018) demonstrated similar spatial patterns in larval dispersal probabilities than predicted by the sea surface SDMs in this study, based on a correlative approach. That both process‐oriented and correlative approaches resulted in similar spatial outcomes, suggests that the absence of data on the distribution of larvae in offshore oceanic areas did not substantially bias sea surface SDM outputs.

Both modeling approaches (correlative SDMs and process‐orientated dispersal models) relied on environmental data collected at the sea surface, thus excluding midwater layers. Spiny lobster larvae (called phyllosomas because of their leaf‐like shape) undertake diel vertical migrations, whereby they rise to surface layers at night to feed and sink deeper during daytime (Bradford et al., 2005). Butler et al. (2011) found that earlier larval stages of *Panulirus argus* occurred almost exclusively in surface waters (0–50 m depth) whereas later stages undertook daily migrations to depths of around 100 m, but they remained present in upper layers. The assumption that sea surface data will represent habitats of drifting larvae adequately was therefore met in the present study, without the additional complexity of modeling midwater layers.

Forward prediction of habitat suitability under climate change scenarios has been undertaken for several spiny and clawed lobster species, using a variety of climate projections and modeling frameworks (Boavida‐Portugal et al., 2018; Caputi et al., 2013; Moya et al., 2017; Tanaka et al., 2019). We used a worst‐case RCP 8.5 scenario (IPCC, 2013; Stock et al. 2011) and an 80‐year horizon to the year 2,100 to maximize contrast at an ocean‐wide geographic scale. Change in highly suitable habitat areas ranged between −33% and +8% in sea surface predictions, and between −20% and −40% in benthic predictions. These results agree with projected high losses of spiny lobster diversity in eastern Africa and the Indo‐Pacific found by Boavida‐Portugal et al. (2018) based on a RCP 4.5 stabilization scenario.

The specific mechanisms driving range shifts under climate change are not yet fully understood, but ocean currents and water temperature are likely to play important roles in larval transport and survival (Cetina‐Heredia et al., 2015; Cowen & Sponaugle, 2009). Using a seascape genetics approach, Singh et al. (2018) highlighted the importance of ocean currents (including steep oceanographic gradients or fronts) and water temperature on the dispersal and genetic structure of *P. homarus*. Changes in ocean current systems and sea surface temperature profiles, predicted under most climate change scenarios (incl. RCP 8.5), are therefore likely to influence spatial dispersal patterns, larval survival, and benthic settlement success of postlarvae (Caputi et al., 2013). An important caveat of the SDMs used in this study is that they do not consider biotic factors or species interactions in predictions of suitable habitats (Hutchinson, 1957; Soberon & Peterson, 2005) but rely only on abiotic environmental conditions, strengthened by information on dispersal capabilities (or movements) obtained from a previous process‐oriented study (Singh et al., 2018).

In conclusion, genetic partitioning of a widespread marine spiny lobster revealed unique local adaptations, which were not apparent in SDMs that excluded the genetic information. Individual subspecies were adapted to distinct combinations of ocean current, water temperature, and salinity regimes. Drifting larvae and benthic life history phases were adapted to different combinations of sea surface and benthic environmental variables considered. Highly suitable habitats for benthic phases were spatially enveloped within sea surface habitats suitable for drifting larvae. SDMs predicted that highly suitable habitats for *P. homarus* subspecies will decrease by the year 2,100, based on the RCP 8.5 climate change scenario.

## CONFLICT OF INTERESTS

The authors declare that there are no competing interests.

## AUTHOR CONTRIBUTION

**Sohana P. Singh:** Conceptualization (equal); Formal analysis (lead); Funding acquisition (supporting); Investigation (equal); Methodology (equal); Project administration (equal); Software (lead); Visualization (lead); Writing‐original draft (lead); Writing‐review & editing (equal). **Johan C. Groeneveld:** Conceptualization (equal); Funding acquisition (lead); Methodology (equal); Project administration (equal); Resources (lead); Supervision (equal); Validation (equal); Writing‐review & editing (equal). **Sandi Willows‐Munro:** Conceptualization (equal); Investigation (equal); Methodology (equal); Project administration (equal); Supervision (equal); Writing‐review & editing (equal).

## Data Availability

Data used in this study are publicly available at https://www.bio‐oracle.org/ , www.gbif.org, and www.iobis.org.
